# Stalking, harassment, gendered abuse, and violence towards politicians in the COVID-19 pandemic and recovery era

**DOI:** 10.3389/fpsyt.2024.1357907

**Published:** 2024-04-16

**Authors:** Susanna Every-Palmer, Oliver Hansby, Justin Barry-Walsh

**Affiliations:** ^1^ Department of Psychological Medicine, University of Otago, Wellington, New Zealand; ^2^ Mental Health, Addiction and Intellectual Disability Services, Te Whatu Ora Health, New Zealand, Capital, Coast and Hutt Valley, New Zealand

**Keywords:** stalking, politicians, fixated threat assessment, violence, mental health, gender, social media, COVID-19

## Abstract

**Background:**

High levels of harassment and threats against parliamentarians are being reported internationally, especially in the social media space. This is occurring alongside changes in our social landscape, with increasing political polarisation and the ongoing ramifications from the COVID-19 pandemic. Harassment of politicians has been shown to have implications for psychological wellbeing and physical safety.

**Objectives:**

To investigate harassment and violence towards parliamentarians in the COVID-19 pandemic and recovery era, including whether there had been a change in its nature and quantity, and to explore the contribution of social media.

**Methods:**

A survey of all New Zealand’s parliamentarians was fielded in 2022, focusing on their experiences of harassment analysed quantitatively and with manifest and latent content analysis of free text responses. Data were disaggregated and compared by gender. Secondary analyses were conducted on similar data collected from parliamentarians in 2014 to compare trends over time.

**Findings:**

We obtained a cleaned achieved survey sample of 54 Members of Parliament (MPs). Harassment was reported by 98% of respondents, ranging from disturbing communication to actual physical violence. The vast majority of MPs endorsed multiple modalities of harassment occurring on multiple occasions. Ninety-six percent of MPs had been harassed over social media, with over half being threatened, including threats of physical violence (40%), sexual violence (14%), threats made towards MP’s family members (19%), threats towards staff (12%), and death threats (27%). Almost all forms of harassment had increased significantly since 2014. Most MPs reported experiencing abuse related to the Government response to the COVID-19 pandemic (e.g. lockdowns and vaccine mandates). Many MPs commented that the frequency and intensity of abuse increased markedly during the COVID pandemic and had not subsequently abated. Women were at significantly higher risk of certain types of social media harassment including gendered abuse, sexualised comments, threat of sexual violence, and threats toward their family.

**Conclusion:**

Harassment of parliamentarians is an escalating issue. Online threats and misogyny are increasingly apparent. This harassment has significant psychosocial costs for victims, their family and staff, and for democratic processes.

## Introduction

Politicians have been targets of harassment and threats since long before the emergence of social media. Owing to their public profiles and the nature of their job, politicians are susceptible to stalking and threats from disgruntled constituents with personal grievances, extreme ideologies or fixated views (1, 2). In comparison to other public figures such as celebrities, politicians are also at greater risk of being physically attacked by those who harass them (3). This small but significant risk has been starkly highlighted over the last decade by fatal attacks on politicians, resulting in the deaths of Jo Cox and David Amess (United Kingdom, 2016; 2021), Walter Lübcke (Germany, 2019), and Shinzo Abe (Japan, 2022).

The emergence of social media has also changed the way politicians and citizens interact, offering a direct channel for communication and engagement. Political parties have been quick to see the advantages of this, with the international literature showing rapid uptake of social media by politicians over the last two decades (4). However, this shift has also enabled new channels for harassment. The anonymity of the digital landscape can embolden some users to engage in aggressive, derogatory, and threatening behaviour, directed towards people with viewpoints that diverge from their own (5, 6). Politicians are ready targets for such harassment, which can manifest as hate speech, threats, personal attacks, doxxing (sharing private information online), and the dissemination of misleading content (7–9).

Studies of Members of Parliament (MPs’) experiences of threatening behaviour have been conducted in multiple jurisdictions including Canada, the UK, Australia, Sweden, Norway, and the Netherlands (8, 10–14). In New Zealand, a previous survey we conducted in 2014 identified 87% of MPs had been harassed (7). This harassment occurred in multiple modalities and had significant associated harm. Half of those who were harassed believed that the people responsible for the harassment had a mental illness. Compared to surveys which had taken place before 2010, the New Zealand survey identified a higher rate of harassment via social media at 60%. This was attributed to the rapid growth in use of social media over the intervening time period. These results provided the impetus for the establishment of Ngā Purapura - the Fixated Threat Assessment Centre New Zealand (FTACNZ), a multidisciplinary collaboration between Health, Police and the Parliamentary Service to provide intervention to manage the threats presented to MPs by fixated people.

Some evidence suggests that the risk of MPs being harassed has changed over recent times. In New Zealand, where the current study is based, media has reported on the increasing concerns MPs have for their safety both generally (15), and for those who perceive that they have become a particular target because of their gender, ethnicity or other factors (16). This is consistent with international trends. An analysis comparing online abuse towards United Kingdom (UK) politicians in the month before the 2015 and 2017 election found a significant increase in abusive content on Twitter (17). A survey exploring the MP’s experiences of ‘trolling’ in the UK (18) found all participants reported online abuse. Male MPs reported a greater volume of online abuse, but women were more likely to experience racial and sexual abuse. The authors observed,

“Male MPs reported more concern about reputational damage, and females more concern about their personal safety. Moreover, the impact of this trolling seemed to have a greater effect on female MPs compared to male MPs”

(18). In a qualitative study of European women MPs and their staff, most of those interviewed had encountered disparaging or sexual images or comments about them on social media (19).

Since the time those studies were conducted, the landscape has been further changed by the COVID-19 pandemic and an associated rise in expression of so-called alt right beliefs and conspiracy theories as such as Q-ANON (20). According to an examination of survey data from eight Western countries (n = 49,116) ‘pandemic fatigue’ increased between 2020 and 2021, engendering significant political discontent, including protest support and conspiratorial thinking (21). New Zealand had a particularly stringent lockdown, with significant associated distress reported in the community (22). During the pandemic, there was concerning evidence of proliferation of violent, misogynist and extreme rhetoric directed towards politicians that seeded on telegram channels and spread across other internet platforms like YouTube, Facebook and Instagram (23). On 9 February 2022, anti-vaccine mandate protestors occupied parliamentary grounds, where they remained for over three weeks until they were forcibly evicted by police in March 2022. Throughout the occupation, some protestors advocated for the public trial and execution of prominent politicians for ‘crimes against humanity’, and the protest ended in a violent riot, with police officers attacked, and public property burnt and damaged. (24, 25).

The subsequent resignation of Jacinda Ardern as Prime Minister of New Zealand in January 2023 prompted speculation that harassment and threats contributed to her decision to leave politics (26, 27). An analysis of anonymous posts about key high-profile figures on social media platforms from 2019–2022 found Ms Ardern had been the subject of online vitriol – posts classified by language analysis programs as negative, hateful, sexually explicit, or toxic – at a rate over 50 times higher than any other person studied (28).

The online vitriol translated into real world safety concerns. There was a marked increase in in threats towards the then Prime Minister and MPs more generally that attracted police attention reported in 2021 (29). From its establishment in July 2019 up until the time of Ms Ardern’s resignation, 53% of all referrals to FTACNZ arose from individuals who either targeted her individually, or as a prominent figure in their concerning and threatening communications (241 of 451 referrals). Further, it is the experience of one of us (JBW, the psychiatry lead for FTACNZ), that since the establishment of FTACNZ, there has been an increase in the seriousness of threats, and in their sexist and racist content toward women and parliamentarians from certain ethnic groups.

Given the documented evidence that the nature of abuse and trolling was changing, we considered it important to explore experiences of harassment with MPs. This included clarifying if there had been an increase in harassment and whether this harassment was causing measurable harm to individuals. From our previous research (7) and the ongoing work of FTACNZ, we appreciated this harm may not be borne solely by the targeted MP but also by their family members and staff. We were also mindful of the previous research findings that those that harass, stalk, petition or otherwise fixate on public figures may themselves incur significant harm, which can also spill over to those around them and their communities (30).

Thus, this survey aimed to establish whether there had been a change in the nature and quality of the harassment of MPs since the previous survey in 2014 and to explore the contribution from social media harassment. We aimed to establish whether there were differences in gender and ethnicity and to assess the extent to which this the harassment of MPs caused harm, physically, psychologically and socially to the MPs and to those close to them.

## Methods

The survey was fielded through Qualtrics, a secure online survey platform which can be used to create customisable templates, question types, and survey logic, and to distribute survey link to eligible participants through email or other channels to facilitate anonymous response. Qualtrics is widely used in the international academic research community and is specifically geared to support online or offline data capture for research studies. We designed the survey to ensure compatibility with mobile phone, tablet or computer access.

Survey questions are available as a **Supplementary File S1**. Demographic information collected included: age band, gender (male, female, gender diverse), ethnicity, band of time as an MP, and whether the participant had ever served as a Minister or Associate Minister. Ethnicity was collected using the NZ Census question on ethnicity, which allows individuals to specify multiple ethnic groups. For analysis, individuals were prioritised into ethnic groups following standard guidelines. The questions about harassment were based on our previous survey conducted in 2014, with additional questions added about social media harassment, some of which were adapted from other research (18). We pre-tested the questionnaire on a small sample of colleagues and further modified it to address respondents’ feedback.

The questionnaire was distributed by an email sent individually to each MP’s official parliamentary email during a week Parliament was sitting. It was sent to 119 MPs (New Zealand usually has 120 MPs, but there was an unfilled resignation at the time the survey was fielded). After viewing the information sheet and consenting to participate, MPs anonymously answered questions about their experiences of different types of harassment. The survey took approximately 8 minutes to complete. It was fielded between 18 October and 28 November 2022. This was approximately two years into a three-year parliamentary cycle and was not during an election year.

### Ethics approval

The study was approved by the University of Otago Human Ethics Committee, and we undertook Māori consultation via the Ngāi Tahu Research Committee. Locality approval was given by Parliamentary Services.

### Data analyses

Relative frequencies were calculated for categorical data and reported as percentages. Percentages reported represent the response to each question and not the total number of participants (participants could skip questions and a small number of participants did not respond to every question). Chi-Square analyses were used to evaluate Tests of Independence using crosstabulation (also known as a bivariate table) for categorical variables such as gender and time periods (2022 versus 2015). For measures where the absolute numbers were low (like ethnicity groups), analysis by group was not possible. Quantitative data preparation and analysis were conducted using SPSS v29.

Free text data were analysed using manifest and latent content analysis (31). Content analysis can capture the meanings within data, including data from questionnaires, and involves establishing categories and identifying the frequency by which they occur (32). Manifest content analysis involves the systematic coding and categorisation of visible and obvious themes, patterns, or meanings that are evident in the text or responses. Latent content analysis involves identifying underlying or implicit meanings, themes, or patterns that are not readily apparent in the text or responses. This involves a more in-depth analysis and interpretation of the data to uncover deeper insights. Where we provide illustrative quotes, parts of the quote may have been redacted to preserve participant anonymity.

## Results

In total, there were 58 survey responses. The non-completion rate, defined as those who opened and started but did not complete the survey before the cut-off time, was 7%. We obtained a cleaned achieved sample of 54 (a response rate of 45%), comprising 20 men and 34 women, an overrepresentation of female participants compared with parliament as a whole, where the gender balance is equal. In terms of prioritised ethnicity, 38 participants (70.4%) were NZ European, 10 (18.5%) Māori, four (7.4%) Pacific Peoples, and two were another ethnicity. Participants ranged in age between being in their twenties and their seventies. Eleven (20.4%) had served as a Minister or Associate Minister.

### The frequency and nature of harassment

Harassment was reported by 98% of responding MPs, ranging from disturbing communication, through to actual physical violence. The vast majority of MPs endorsed multiple modalities of harassment occurring on multiple occasions. Harassment over social media and harassment by letters and emails were the two most common forms of harassment (both experienced by 96%), followed by public approaches (82%), distribution of malicious material (73%), and alarming behaviour at the electorate office (62%). The type and frequency of the different forms of harassment is shown in **Table 1**. Compared with 2014, when we previously surveyed politicians on their experiences of harassment, almost all forms of harassment had increased.

**Table 1:** Percentage of MPs experiencing different forms of harassment in 2014 and 2022.

	Number reporting experience/total responses in 2022	Percentage reporting experience in 2022	Percentage reporting experience in 2014	Difference between 2022 and 2014 p-value
Inappropriate social media contact	52/54	96%	60%	>0.01
Inappropriate letters, faxes or emails	50/52	96%	68%	>0.01
Inappropriate telephone calls	29/52	56%	45%	0.19 (NS)
Alarming behaviour at electorate office	31/50	62%	62%	0.76 (NS)
Unwanted approaches	42/51	82%	50%	>0.01
Distribution of malicious material	38/52	73%	48%	>0.01
Threats to harm	33/52	63%	48%	0.04
Loitering	23/52	44%	28%	0.05
Following behaviour	21/50	42%	22%	>0.01
Property interference	22/51	43%	31%	0.15 (NS)
Spurious legal action	10/51	20%	11%	0.14 (NS)
Physical attack (actual or attempted)	9/51	18%	15%	0.14 (NS)

Content analysis of the free text descriptions of the nature and context of this harassment generated three overarching themes. First, participants described a ‘*changing landscape’* with racist, misogynistic, and extreme right rhetoric proliferating online over the preceding two years, which frequently expressed overtly violent ideation toward politicians. They felt this online discourse had emboldened threatening confrontations that occurred in person. The protest movement against COVID-19 restrictions was frequently cited as a watershed event in escalating the hostility against the Government and its representatives. Second, was the theme of *‘fear*’ in which participants described pervasive anxiety that they or someone close to them might be attacked and seriously hurt (or killed). Lastly, participants identified ‘*inadequate support’*, feeling that resources available in terms of security, training, cybersafety and specialist supports had not kept pace with the changing landscape and furthermore were often not available to their staff who were often at the coalface of the abuse.

Quotes from the free text responses illustrating these themes appear in the following paragraphs to provide richness to the quantitative data.

### Location of harassment

Harassment commonly occurred at Parliament, the electorate office and online, but MPs reported they had been targeted at a wide variety of public places. Free text comments described a variety of disturbing and threatening approaches in public, which caused MPs to fear for the safety of themselves and those close to them.

“Intimidating protestors surrounded building and then car I was travelling in.”

“Followed in car around town. Had to wait in public lit place until car left and had dialled 111. The driver was yelling racial, homophobic and other insults whilst tailgating. Not isolated event.”

“A group of people were opposed to a policy I proposed as Minister, and they confronted me at a meeting in my hometown. Following the meeting I received death threats … The police were involved and I had to use a GPS tracker 24/7 … my children were living at home and they were distressed about this and my partner didn’t want me going anywhere on my own. I had difficulties sleeping for a while.”

Actual or attempted attacks were reported by 18% of MPs and almost half (48%) had been threatened with physical harm.

“Someone came to electorate office wanting to stab me. Police intervened.”

“I was assaulted on the way to work.”

“I was told I was responsible for the deaths of [redacted, not COVID related] and I would be captured and hung as a result.”

Property violations were relatively common, being reported by 43% of MPs. Several MPs reported attacks on their homes.

### Social media

Being harassed over social media was experienced by 96% of participants, reported as occurring daily or weekly by 68%. The type of abuse MPs received included racial abuse, gendered abuse, sexualised comments, and abuse related to sexual orientation (**Table 2**). Almost six in ten participants had been abused regarding the restrictions aimed at combating COVID-19. Over half of MPs had been threatened over social media. These threats included threats of physical violence, sexual violence, threats made towards MP’s family members, threats made towards staff, and death threats.

**Table 2:** Percentage of MPs experiencing different types of abuse over social media compared by gender.

	Male (n=20)	Female (n = 32)	Total (n = 52)	Chi-squared test p-value
Racial abuse	25.0%	33.3%	30.2%	0.41 (NS)
Gendered abuse	25.0%	62.5%	48.1%	<0.01
Sexualised comments	10.0%	40.6%	28.8%	<0.05
COVID-related abuse	50.0%	62.5%	57.9%	0.37 (NS)
Sexual orientation abuse	5.0%	15.6%	11.5%	0.24 (NS)
Threats of physical violence	30.0%	46.9%	40.4%	0.23 (NS)
Threats of sexual violence	0%	21.9%	13.5%	<0.05
Threats to family	5.0%	28.1%	19.2%	<0.05
Threats to staff	5.0%	15.6%	11.5%	0.24 (NS)
Death threats	15.0%	34.4%	26.9%	0.13 (NS)
Abuse on political grounds	95%	81.3%	86.5%	0.16 (NS)
Abuse on religious grounds	20%	15.6%	17.3%	0.68 (NS)
Threats of reputational damage	50.0%	46.9%	48.1%	0.82 (NS)

MPs generally felt the frequency of social media harassment had increased over the last two years with 73% of participants reporting the number of abusive messages/threats they received over social media had increased (19% said they had not increased and 8% said they had decreased).

Harassment over social media often escalated into in person confrontations, with MPs reporting that their harassers had approached them in public (35%), or even turned up at their home (9%). Over half of MPs (53%) blocked people from their social media account.

### Gender

As previously mentioned, a theme from the free text content analysis was an increase in abuse that was overtly misogynistic and/or racist. Statistical analysis of the quantitative data found women were at significantly higher risk of reporting certain types of social media harassment, including gendered abuse, sexualised comments, threat of sexual violence, and threats toward their family (**Table 2**). Support for transgender rights was also seen as attracting a lot of vitriol.

“There was a focus on my gender - I found it more rattling than I expected.”

“Had [my electorate] office tagged with misogynistic and anti-trans messages.”

There was a trend of women being more likely to receive death threats over social media (34% of women had experienced this compared to 15% of men) but this did not reach statistical significance. Abuse on political grounds was high for both genders but was reported by 95% of men compared to 81% of women.

### COVID-related abuse

Fifty-eight percent of MPs reported experiencing abuse related to the COVID-19 pandemic and the related Government response. A strong theme that came through in the free text comments was that the frequency and intensity of abuse had increased during the course of the anti-vaccine and anti-mandate occupation of parliament in February–March 2022, but this intensity had not subsequently subsided as many had hoped.

“[Harassment] has increased significantly, primarily driven by Covid conspiracism. As it stands, I think it likely someone may be seriously hurt.”

“Covid seems to have exacerbated the volume and nastiness of abuse. I had thought it might start decreasing by now, but it seems to have kept up.”

“Sitting in my office during the protests hearing people saying MPs should be killed day after day was stressful, even though I refused to stop coming to work. I watched the riots happen and it was freaky, knowing some of those people would likely take their violence out on an MP.”

### Impact of harassment

The impact on those harassed included fearfulness for their own safety (reported by 72%), a reduction in social outings (40%), concern being alone at home (24%), a change in daily routines (41%), a change in personal relationships (18%) and lost time from work (10%). The deleterious consequences of this harassment on MPs’ professional and personal lives had increased since 2014 (**Table 3**). In 2014, half the MPs reported having ever felt a degree of fear (this rose to 60% when considering the subgroup who had experienced harassment), but then only 20% had ever felt that their personal safety might be in jeopardy, compared to 72% in 2022. Some longstanding MPs reflected that they perceived the risks having changed substantially in the course of their careers.

**Table 3:** Impact of harassment on MPs in 2014 and 2022.

Response/consequences of harassment	2022 (%)	2014 (%)	p-value
Increase security at home	64%	25%	<0.01
Increase security at work	51%	34%	0.09 (NS)
Change telephone number	8%	5%	0.61 (NS)
Concern going out in public	40%	12%	<0.01
Concern being home alone	24%	11%	<0.01
Change routine	41%	10%	<0.01
Reduce outings	40%	12%	<0.01
Change in personal relationships	18%	9%	0.13 (NS)
Lost time from work	10%	4%	0.19 (NS)
Fearful for own safety	72%	20%	<0.01
Staff/family fearful	80%	28%	<0.01
Mental or emotional stress	62%	[Not asked]	–
Problems with friends or family	19%	[Not asked]	–
Damage to reputation	38%	[Not asked]	–
Problems at work	28%	[Not asked]	–

“Earlier in my career I prided myself on having a policy of accessibility and openness. Now I fear for my safety and the safety of my staff and have set up security measures at home and on site where my electorate staff operate.”

“The list is exhausting – it’s debilitating and sadly worse than when I first started.”

“I’ve been doing public facing politics for [a long time]. I’ve never, ever seen the tone of public discourse and the emboldening of aggressive, fringe conspiracy thinking like has occurred in the last six months…”

Women MPs in general were more likely to experience feeling unsafe. For example, as a result of threatening or abusive messages on social media specifically, 69% of female and 33% of male participants felt fearful for their safety (p<0.05) and 46% of female and 5% of males felt unsafe in their own homes (p<0.005).

“My red alert safehub didn’t work, the Police were significantly outnumbered and would have been unable to support me - and I had no safe exit. I got out of the situation safely, but I was petrified.”

A number commented in free text responses that their families and staff were more affected than they were.

“I’m fine to stand my ground, but my family and partner are terrified about me now going to events and things by myself.”

MPs worried about safety of those close to them such as partners, children and staff.

“My husband was physically attacked by someone launching at me in public. I have had security put up because I had abusive people turn up to threaten me, we’ve had graphic videos threatening ourselves and whānau [family].”

“… A local person was touting on Facebook claiming to know where I lived seeking to collect a group to go throw rocks at my windows that evening when I knew the only person there that evening was my [teenage] daughter…”

“The threats and harassment to my [redacted]-year-old daughter [were the worst]. She was subjected to social media trolling and harassment because I am her mother.

“Staff member threatened and [the perpetrator] is in jail awaiting trial — [they] threatened to bomb my office. Staff very shaken by this.”

“As a Minister [the most serious episode was] learning that departmental staff were being harassed by phone and personally, that departmental vehicles had been tampered with putting staff safety at risk, and that security had to increase at departmental offices because of intimidating and threatening behaviour by [redacted] individuals who had ready access to firearms.”

Some wanted to acknowledge that although there were risks to their job, many other professions carried similar levels of risk.

“We are elected to represent. That means our jobs can be difficult, but no more so that a police officer or paramedic, for example.”

“MPs need to harden up. They are in no greater danger of actual harm than people who work in, say, forestry, then recognise they have a responsibility to serve the public instead of complaining about them.”

Several MPs emphasised that although their harassers had challenging behaviour, the contact could represent an opportunity for intervention.

“Many of the people who are regarded as harassing us are actually people who require help, which we try to give.”

“Emails are threatening but clearly show this person’s mental state. At once stage I had to ask the support agency to contact him as I believed he was homeless and without food for several days.”

Sixty-four percent of MPs had increased their security at home and 51% had increased their security at work. Some reported sadness at becoming less available to their constituents.

“It is sad for me that this has to happen, because as MPs we are representatives of our community, and the community should be able to access us freely.”

MPs often reported that the harassment they experienced took its toll on their wellbeing and personal life and that this could be a heavy burden to carry.

“It’s been awful and has been detrimental to my physical and psychological and emotional health but more importantly it has detrimentally affected my family relationships … [my children] have as a result distanced themselves … to protect and shield themselves. That has been extremely difficult.”

“It is wearying to routinely experience harassment because of who I am and the Party I stand for.”

### Sources of support

Participants had sought assistance from different sources including the Parliamentary security enablement team (56%), the Police (48%), a private security company (28%), and the Fixated Threat Assessment Centre (26%). Eight MPs (16%) reported seeking help from a health professional. MPs felt there was insufficient support to meet an increased risk and that additional supports should also be available to staff.

“The security response from parliament … is poor with little meaningful support provided given the enhanced risk.”

“The consistent message is that we are significantly vulnerable, and insufficient resources and effort is put into making and keeping us safe. It is not reassuring to hear colleagues talk of ‘not if but when’ an MP is seriously harmed, and that when this happens, there will be a report commissioned that says exactly what we have been saying over and over to deaf ears for far too long.”

“Moderating social media can be draining and very unpleasant for staff who help MPs do this. It can have a negative and stressful effect on them having to regularly see hateful comments, respond to some of them; and block or hide others. These staff are often relatively young and being exposed to an overload of negative comments can affect their mental wellbeing.”

*“It would be good to get more support for staff and AI tools to moderate social media. Knowing that others are monitoring social media to identify extremists helps. So would tools and training around the use of language to help avoid using triggering language.”*

## Discussion

This study showed high rates of harassment that was impacting on the wellbeing of politicians, their staff and families. Harassment was reported by 98% of participants, ranging from disturbing communication to actual physical violence. The vast majority of MPs endorsed multiple modalities of harassment occurring on multiple occasions. Women were at significantly higher risk of certain types of social media harassment including gendered abuse, sexualised comments, threat of sexual violence, and threats toward their family. The qualitative data emphasised the disturbing nature of some of the harassment, including implicit and explicit threats to MPs and their families. The nature of the harassment appeared to be driven by a combination of personal grievances, extremist ideologies, and mental health challenges.

Almost all forms of harassment had increased significantly since 2014. It was not just the frequency of threats that had increased, but their psychological impact. In 2014, MPs were often phlegmatic when talking about their experiences of harassment, reporting a lower degree of distress than expected (7). The 2022 survey found a marked increase in harms arising from harassment/trolling, for example, feeling fear about personal safety had risen by a factor of three. Additionally, nearly two thirds of the participants reported mental or emotional stress, and staff and families were also affected. These findings speak to the high cost of holding public office and the risk to vital democratic activity are similar to those found in the UK (18) and are consistent with the described pernicious psychological effects of sustained harassment (33). Previous research has raised the spectre of harassment and threats to change in the behaviour of elected representatives to lessen engagement with their communities or to decide not to continue in politics if they are subject to harassment and abuse (17, 18, 34). If this is the case, then the damage to our democratic institutions may be considerable. This seems to be playing out in the international reports of online abuse and threats of violence deterring elected representatives from participating in politics, especially for women (35–37).

Fifty-nine percent of MPs reported experiencing abuse related to the Government response to the COVID-19 pandemic, many considering this to be a watershed event in terms of the online vitriol towards parliamentarians that ensued, which then spilled into actual violence during the 2022 occupation of parliamentary grounds. Participants felt the level of the abuse that intensified over the COVID pandemic had not abated at the time of the survey, despite the removal of the restrictions that had provoked the escalation of online hostility.

The online environment with its apparent anonymity, invisibility, and perceived lack of consequences may result in an ‘*online disinhibition effect*’ (5). The online disinhibition effect refers to the phenomenon wherein individuals engage in behaviours on the internet that they might not typically display in face-to-face interactions. This may embolden some individuals to engage in harassment online, even though they would not have behaved that way in person.

Overall, the current political climate, characterised by heightened polarisation and the amplification of extreme viewpoints through digital platforms, seems to be exacerbating threats against politicians. Internationally there is evidence that support for violence to achieve political ends is increasing amongst Western democracies. For example, in a 2023 American study, 38% of Republican supporters agreed violence was acceptable as a means to stop political opponents from reaching their goals (38).

The emergence of disaffected online communities can attract and validate fixated individuals who align with extremist ideologies, leading to intensified online harassment and real-world threats. Notwithstanding the fact that harassment and stalking causes considerable psychological harm, irrespective of any physical violence that may eventuate (33, 39), for many participants, the fear of being hurt or killed, or having a family member harmed by a fixated individual was a live concern.

Concerningly, in our study, it was not just online abuse which had increased, but also threatening approaches toward politicians, which were reported by four out of five politicians and one in five had been attacked.

### Strengths and limitations of this study

The strengths of the study include the repetition of many of the questions used in a 2014 survey of New Zealand MPs, allowing comparison over time. The wealth of free text data provided important insights that could be analysed qualitatively and triangulated with the quantitative data.

However, the study methodology also has obvious limitations. The study design was an anonymous survey, meaning we could not identify the people subjected to harassment or those perpetrating the harassment. Actual harassing communications were not analysed. The threshold of what constituted harassment was subjective, with the concept generally operationalised in the survey by asking participants whether they had experienced abusive or threatening contacts. The sample size was relatively small and the response rate was below 50%, raising the risk of selection bias, although it is to be noted that the response rate is similar to, or exceeds, the rates from similar international studies.

### Future actions and research priorities

It is not appropriate to just monitor and report on this issue. MPs were clear that they and their staff require support and resources to manage these threats. This necessitates a multi-pronged approach and expansion of existing services. For new politicians, de-escalation, safety and cybersecurity training should be part of the induction package, and resources made available to increase home and office security measures. MPs experiencing harassment benefit from easy and rapid access to specialist advice and support. Provision of such services requires not only resource but also regular education and liaison with affected MPs and their staff, from specialist services. In the demanding, fluid and time poor environment MPs work in this can be difficult to deliver. In New Zealand at least, the Government appears increasingly cognisant of this issue, with the public service budget in 2023 containing additional funding earmarked for ‘enhancing security measures for MPs and their staff’.

In addition to MP-centred interventions, early identification and risk assessment of fixated individuals, including thorough background checks and monitoring of online behaviour, can aid in proactive intervention. Collaboration between law enforcement agencies, mental health professionals, and political entities can be effective in identifying, assessing and managing potential threats. In New Zealand, the establishment of FTACNZ, and the roll out of mental health co-response teams involving police, paramedics and mental health professionals responding in tandem, are both good examples of interagency approaches (40–42) but it is evident from our research that more could be done.

Moderation of the online environment is a thorny issue. There needs to be greater engagement with technology companies and the development of algorithms that can quickly identify and remove dangerous content, but achieving this is not simple. One initiative aimed at advancing this is the “Christchurch Call.” This originated in 2019 when the New Zealand Prime Minister and French President brought together political and technology sector leaders in a commitment to work toward eliminating violent extremist content online, after the horror of the live-streamed fatal shooting at two Christchurch mosques (43). This work continues, but complex issues such as balancing free speech and security, and achieving consensus and cooperation among diverse nations and corporations with different legal systems, cultural norms, and priorities, means that progress has been slow.

In terms of future research, factors such as age, gender and ethnicity should be examined in more depth. Our sample was too small to be able to consider ethnicity or age as predictive variables, although we showed that women were at higher risk of concerning harassment such as threats toward their families and being threatened with sexual violence. Other research suggests racism and sexism are common drivers of abuse of politicians. For example, an analysis of Twitter abuse against female MPs in the United Kingdom found in the run up to the 2017 election, nearly half of the abusive tweets were directed at Diane Abbott, the first black woman to be elected to the British parliament. When Abbott was excluded from the sample, black and Asian women still received 30% more abuse than their white colleagues (44).

The survey was fielded shortly after the majority of the COVID pandemic restrictions had ended (including vaccine mandates and mask wearing requirements except in healthcare facilities) (45). There was little doubt in many of our participants’ minds that the pandemic and the Government response to this had contributed to an increased frequency and intensity of abuse. Repeating the survey in a couple of years’ time to track the trajectory of abuse over time is advised.

## Conclusions

Harassment of parliamentarians is an increasingly serious issue. Almost all MPs reported harassment, often via multiple modalities and on multiple occasions. Women were at significantly higher risk of certain types of social media harassment including gendered abuse, sexualised comments, threat of sexual violence, and threats toward their family. Almost all forms of harassment had increased significantly since this survey was last fielded in 2014. Overall, the extent and medium by which this harassment occurs is shifting. This harassment has significant psychosocial costs and requires a multi-pronged response.

## Data availability statement

The datasets presented in this article are not readily available because of the sensitive and confidential nature of the raw dataset. Data will only be made available to researchers with approval of the University of Otago Ethics Committee. Requests to access the datasets should be directed to humanethics@otago.ac.nz and susanna.every-palmer@otago.ac.nz.

## Ethics statement

The studies involving humans were approved by University of Otago Ethics Committee. The studies were conducted in accordance with the local legislation and institutional requirements. The participants consented to participate in this study.

## Author contributions

SE-P: Conceptualization, Data curation, Formal Analysis, Investigation, Methodology, Project administration, Resources, Software, Writing – original draft, Writing – review & editing. OH: Conceptualization, Data curation, Formal Analysis, Investigation, Methodology, Project administration, Resources, Software, Writing – original draft, Writing – review & editing. JB-W: Conceptualization, Data curation, Investigation, Methodology, Project administration, Resources, Software, Writing – original draft, Writing – review & editing.
