# Supplementary material for: Stalking, harassment, gendered abuse, and violence towards politicians in the COVID-19 pandemic and recovery era
Source: Front Psychiatry. 2024 Apr 16;15:1357907. doi: 10.3389/fpsyt.2024.1357907 (PMC11060435; doi:10.3389/fpsyt.2024.1357907)
Supplement: Supplementary file 1 [file Table_1.docx]

Survey of MPs

*Note: This is an unformatted version of the survey questions for the study on stalking and harassment on New Zealand politicians in the COVID-19 pandemic and recovery era. Participants saw a formatted version that was hosted on the Qualtrics platform.*

Start of Block: Default Question Block

Q1 Thank you for taking the time to participate in this survey. It will take between 5 and 10 minutes to complete. Before continuing, please ensure that you have read the Participant Information Sheet attached to the email with the survey. Your participation in the survey is implied consent.

| Page Break |  |
| --- | --- |

Q2  Which ethnic groups do you identify with?  
 ***Please tick****ALL* ***the ethnic groups you identify with.***

- New Zealand European (Pākehā) (1)
- Māori (2)
- Pacific Peoples (3)
- Asian (4)
- Other (5) __________________________________________________

| Page Break |  |
| --- | --- |

Q3 How do you describe yourself?

- Male (1)
- Female (2)
- Non-binary / third gender (3)
- Prefer to self-describe (4) __________________________________________________
- Prefer not to say (5)

| Page Break |  |
| --- | --- |

Q4 How old are you?

- 20–29 years old (2)
- 30–39 years old (3)
- 40–49 years old (4)
- 50–59 years old (5)
- 60–69 years old (6)
- 70 or older (7)

| Page Break |  |
| --- | --- |

Q5 What is the total number of years you have been an MP (cumulatively over your life time)?

- 0–5 years (1)
- 6–10 years (2)
- 11–15 years (3)
- 16–20 years (4)
- Over 20 years (5)

Q6 Have you ever served as a Minister or Associate Minister in Government?

- Yes (1)
- No (2)

| Page Break |  |
| --- | --- |

Q7 **We would like to focus first on harassment over social media (e.g., Twitter, Facebook, Instagram, etc).**

 How often have you received abusive or threatening messages on social media as a result of your role as an MP?

- Daily (1)
- Weekly (2)
- Monthly (3)
- Several times a year (4)
- Once a year or less frequently (5)
- Never (7)

Skip To: Q19 If We would like to focus first on harassment over social media (e.g., Twitter, Facebook, Instagram,... = Never

| Page Break |  |
| --- | --- |

Q8 Please identify the types of abusive or threatening messages that you have received (select all that apply):

- Racial abuse (1)
- Gendered abuse (e.g. abusive messages with gendered content or including gendered slurs) (2)
- Sexualised comments (3)
- COVID-related abuse (4)
- Abuse related to your sexual orientation (5)
- Threats of physical violence (6)
- Threats of sexual violence (7)
- Threats to family (8)
- Threats to staff (9)
- Death threats (10)
- Abuse on political grounds (11)
- Abuse on religious grounds/beliefs (12)
- Threats of reputational damage (13)

| Page Break |  |
| --- | --- |

Q9 Has the number of threats/ abusive message changed over the last two years?

- No, they have not changed (4)
- Yes, they have decreased (1)
- Yes, they have increased (2)

| Page Break |  |
| --- | --- |

Q10 Which of the following applies to how you deal with abusive/ threatening messages over social media?

- I try not to read them (1)
- I read but never respond to them (2)
- I read and sometimes respond to them (3)
- I read and regularly respond (4)
- Other (please specify) (5) __________________________________________________

Q11 Do you block people from your social media?

- Yes (1)
- No (2)
- Sometimes (3)

| Page Break |  |
| --- | --- |

Q12 To the best of your knowledge, has anyone who has abused or threatened you on social media also abused or threatened you in other ways (e.g., phone calls, email, approached you in person)? Please select all that apply:

- No, not to the best of my knowledge (1)
- Possibly, but I'm not sure (2)
- Yes, by email (3)
- Yes, by phone (4)
- Yes, approached me in a public place (5)
- Yes, turned up at my home (6)
- Other. Please describe: (7) __________________________________________________

| Page Break |  |
| --- | --- |

Q13 As a result of receiving threatening or abusive messages via social media, did you ever:

|  | No (1) | Yes (2) |
| --- | --- | --- |
| Increase your security at home (1) |  |  |
| Increase your security at work (2) |  |  |
| Change your phone number (3) |  |  |
| Take time off work (4) |  |  |
| Reduce your social outings (5) |  |  |
| Change your daily routines (6) |  |  |
| Experience changes in your close relationships (7) |  |  |
| Feel concerned about going out in public (8) |  |  |
| Feel concerned about being home alone (9) |  |  |
| Feel fearful for your safety (10) |  |  |
| Feel fearful for the safety of those close to you (11) |  |  |
| Feel frightened that you may be physically assaulted (12) |  |  |

| Page Break |  |
| --- | --- |

Q14 Did receiving threatening or abusive messages via social media ever cause you any of the following (select all that apply):

- Mental or emotional stress (1)
- Problems with your friends or family (2)
- Damage to your reputation (3)
- Problems with romantic relationships (4)
- Problems at work (5)
- Other (please specify) (6) __________________________________________________
- Prefer not to say (7)

| Page Break |  |
| --- | --- |

Q15 As a result of receiving threatening or abusive messages via social media, have **you** ever felt frightened or fearful?

- Not at all (1)
- Only a little or somewhat fearful (2)
- Moderately fearful (3)
- Very fearful (4)

Q16 As a result of receiving threatening or abusive messages via social media, have any of your **family members** ever felt frightened or fearful?

- Not at all (1)
- Only a little or somewhat fearful (2)
- Moderately fearful (3)
- Very fearful (4)

Q17 As a result of receiving threatening or abusive messages via social media have any of your **staff** ever felt frightened or fearful?

- Not at all (1)
- Only a little or somewhat fearful (2)
- Moderately fearful (3)
- Very fearful (4)

| Page Break |  |
| --- | --- |

Q18 As a result of receiving threatening or abusive messages via social media, did you ever seek help or advice from any of the following:

|  | No (3) | Yes (4) |
| --- | --- | --- |
| Family, friends or work colleagues (9) |  |  |
| Police (10) |  |  |
| The security enablement team (11) |  |  |
| A lawyer (12) |  |  |
| A health professional (13) |  |  |
| Security company (14) |  |  |
| The Fixated Threat Assessment Centre (15) |  |  |
| Other, please specify (16) |  |  |

| Page Break |  |
| --- | --- |

Q19 **Now we would like to ask about harassment *other* than via social media.**

 Related to your role as an MP, approximately how often have you experienced the following over the course of your career:

|  | Never (1) | Once (2) | Twice (3) | More than twice, but less than ten times (4) | Ten or more times (5) |
| --- | --- | --- | --- | --- | --- |
| Been attacked or have someone attempt to attack you (1) |  |  |  |  |  |
| Been personally threatened or have someone close to you threatened, either directly or indirectly (2) |  |  |  |  |  |
| Received unwanted approaches (e.g. at home, at work, in public places) (3) |  |  |  |  |  |
| Had someone behave in an alarming way at your electorate office (4) |  |  |  |  |  |
| Been followed (on foot, by car) (5) |  |  |  |  |  |
| Had someone loitering around your home or public places that you frequent (6) |  |  |  |  |  |
| Had someone interfere with your property (e.g., car) (7) |  |  |  |  |  |
| Had someone initiate spurious legal action against you (8) |  |  |  |  |  |
| Had someone distributing malicious material about you (9) |  |  |  |  |  |
| Received inappropriate correspondence (e.g., letters, emails, text messages) (10) |  |  |  |  |  |
| Received inappropriate telephone calls (11) |  |  |  |  |  |

| Page Break |  |
| --- | --- |

Q20 If you have been threatened or harassed at different times and by different people, please refer to the harassment that **felt** **the most serious**.

 Are you able to briefly describe what happened and how this affected you and/or the people around you?

________________________________________________________________

________________________________________________________________

________________________________________________________________

________________________________________________________________

________________________________________________________________

Q21 Was the identity of the harasser/s (from the previous question) known?

- Yes, they were identified but were a stranger to me (1)
- Yes, they were someone I knew or knew of (2)
- No, the person was never identified (3)

| Page Break |  |
| --- | --- |

Q22 Have you ever been threatened or harassed by someone you suspected was mentally unwell?

- Yes (1)
- No (2)
- I have no idea (3)

Q23 Can you describe what happened?

________________________________________________________________

________________________________________________________________

________________________________________________________________

________________________________________________________________

________________________________________________________________

| Page Break |  |
| --- | --- |

Q37 You are coming to the end of the survey. Before you finish, is there anything else about this topic that you would like to say?

________________________________________________________________

________________________________________________________________

________________________________________________________________

________________________________________________________________

________________________________________________________________

| Page Break |  |
| --- | --- |

Q24 If this survey has raised any distress or concerns you wish to further discuss, you can contact the lead investigator, Dr Justin Barry-Walsh at justin.barry-walsh@police.govt.nz/. Justin is a psychiatrist with expertise in this area who will be able to provide support and advice. Justin also works for the Fixated Threat Assessment Centre.

 The Fixated Threat Assessment Centre can also take referrals directly in relation to experiences of stalking or harassment you have had. They can be contacted at ftac@police.govt.nz

End of Block: Default Question Block
